# P-1297. Nocardia Infections in Oncology Patients: Risk Factors and Patterns of Resistance

**DOI:** 10.1093/ofid/ofaf695.1485

**Published:** 2026-01-11

**Authors:** Anushi Patel, Cilia Nazef, Shivan Shah, Ana Velez, Guy Handley, Yanina Pasikhova

**Affiliations:** University of South Florida, Tustin, CA; University of South Florida, Tustin, CA; University of South Florida, Tustin, CA; University of South Florida, Tustin, CA; The University of Texas MD Anderson Cancer Center, Houston, Texas; Moffitt Cancer Center, Tampa, Florida

## Abstract

**Background:**

*Nocardia* can lead to disseminated disease with high mortality in immunocompromised patients. Though *Nocardia* has been well documented in solid organ transplant recipients, its impact on patients with malignancies without bone marrow transplantation is less characterized.

*Nocardia* species exhibit varying susceptibility to antibiotics and treatment requires prolonged courses of multiple antibiotics [1-3]. As our cancer population continues to grow, we are seeing increased cases of nocardiosis, urging the need for better treatment strategies. We investigated the epidemiology, risk factors, antimicrobial susceptibility, and outcomes of oncology patients with proven nocardiosis over a 15-year period.

**Figure:**
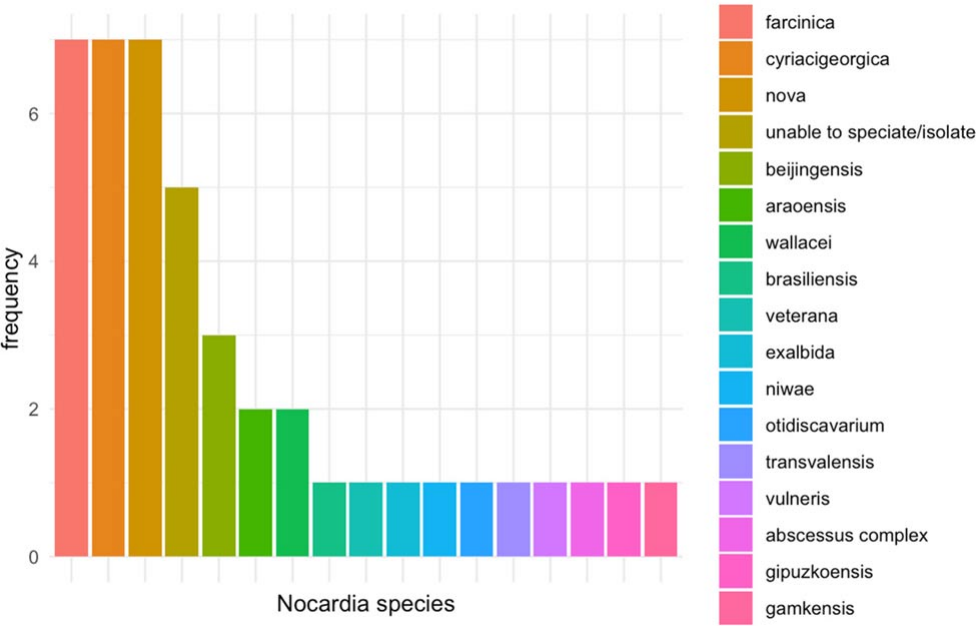
Nocardia species

**Figure:**
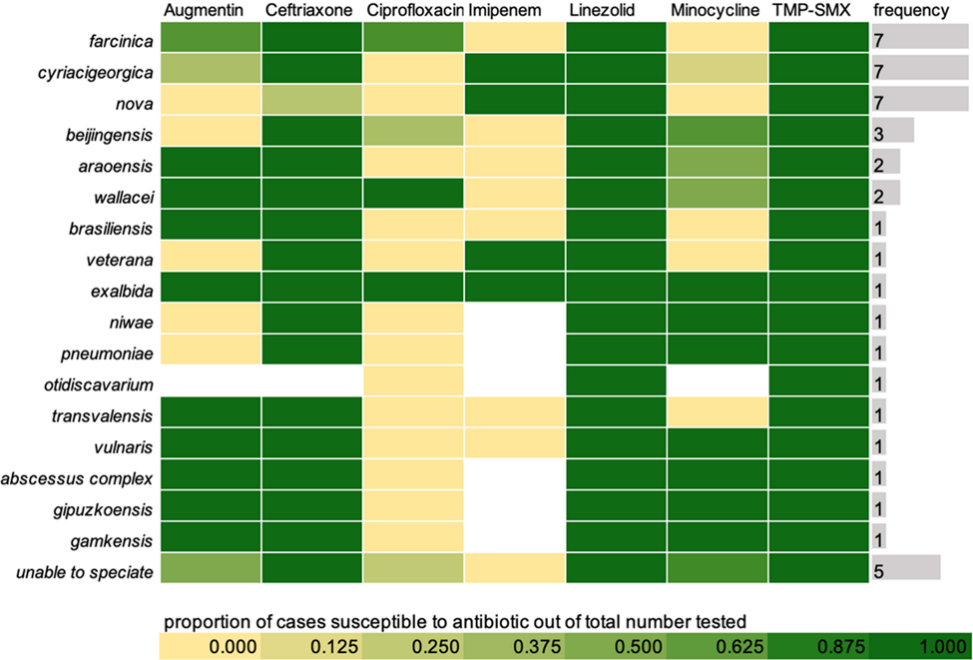
Heat Map description of the varying identified species and their susceptibilites

**Methods:**

This retrospective single-center cohort study reviewed all patients with nocardiosis from 2010 to 2025. In total, 40 patients were identified. Demographics, comorbidities, clinical presentations, epidemiology, susceptibility patterns, and outcomes were evaluated and described.

**Figure:**
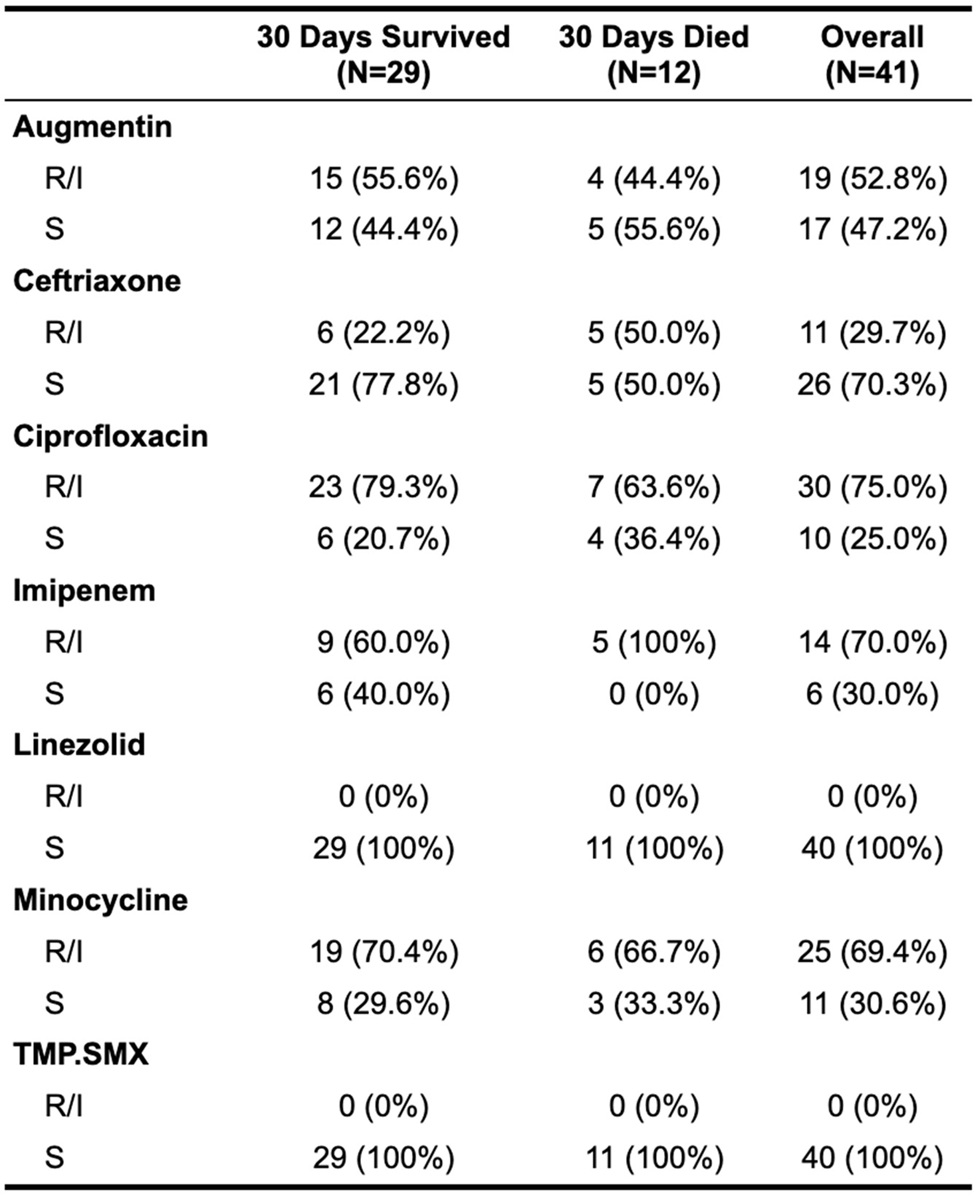
Nocardia susceptibilities

**Figure:**
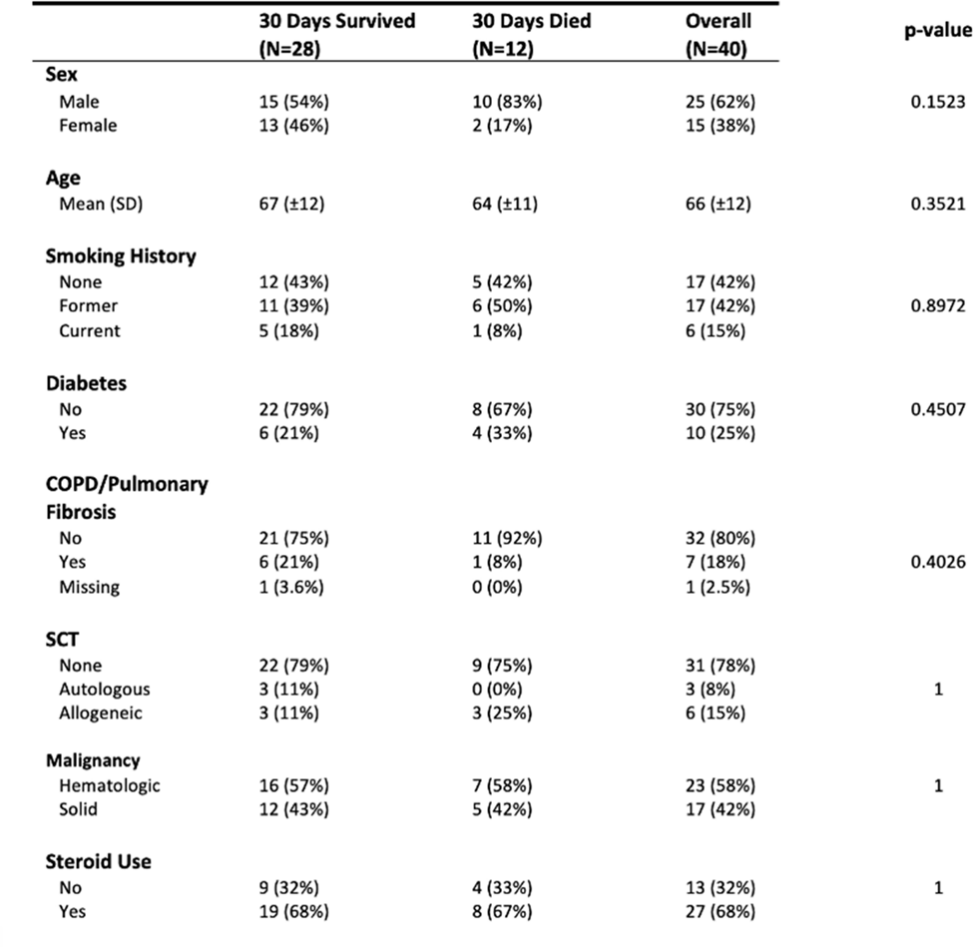
Risk Factors and 30 Day Mortaility

**Results:**

By 30 days after positive culture, 12/40 (30%) patients had died. No association was found between sex, age, type of malignancy, degree of dissemination, initial antimicrobial regimen or medical comorbidities evaluated. In total, 16 unique *Nocardia* species were identified, most frequent were *N. farcinica* , *N. cyriacigeorgica* , and *N. nova.* All organisms exhibited susceptibility to linezolid and trimethoprim-sulfamethoxazole (TMP-SMX); of those tested for imipenem, 14/20 isolates (70%) demonstrated resistance.

**Conclusion:**

Our findings demonstrate the diversity of pathogenic *Nocardia* species, antibiotic resistance patterns, and the high 30-day mortality rate (30%) from these infections. All species were susceptible to linezolid and TMP-SMX, while a sizable portion were resistant to imipenem. Empiric regimens may consider linezolid or TMP-SMX, while the role of imipenem may need to be further evaluated.

**Disclosures:**

All Authors: No reported disclosures

